# The emerging roles of ribosomal histidyl hydroxylases in cell biology, physiology and disease

**DOI:** 10.1007/s00018-018-2903-z

**Published:** 2018-08-27

**Authors:** James R. Bundred, Eline Hendrix, Mathew L. Coleman

**Affiliations:** Tumour Oxygenase Group, Institute of Cancer and Genomic Sciences, University of Birmingham, Edgbaston, Birmingham, B15 2TT UK

**Keywords:** Histidine, Hydroxylation, Post-translational modification, Cancer, Bone development, Immunology

## Abstract

Hydroxylation is a novel protein modification catalyzed by a family of oxygenases that depend on fundamental nutrients and metabolites for activity. Protein hydroxylases have been implicated in a variety of key cellular processes that play important roles in both normal homeostasis and pathogenesis. Here, in this review, we summarize the current literature on a highly conserved sub-family of oxygenases that catalyze protein histidyl hydroxylation. We discuss the evidence supporting the biochemical assignment of these emerging enzymes as ribosomal protein hydroxylases, and provide an overview of their role in immunology, bone development, and cancer.

## Introduction

Post-translational modification (PTM) is a major level of protein control that plays critical roles in all fundamental cellular processes. Our understanding of the proteomic landscape of PTMs and the discovery of new protein modifications has been partly fuelled by the development of mass-based detection, sequencing and quantification technologies. Mass spectrometry has revolutionized the ability to research small and difficult-to-detect PTMs. One such PTM is ‘hydroxylation’, the enzymatic incorporation of a single oxygen atom to create an alcohol, or hydroxyl, group. Although hydroxylation was once considered to be a very rare PTM [1], it is now appreciated to be an important modification that deserves more scrutiny for its roles in biology and for its potential medical applications [2].

Protein hydroxylation is generally catalyzed by a family of ‘2-Oxoglutarate (2OG)-dependent oxygenases’ (2OG-oxygenases) (see below). These enzymes utilize fundamental nutrients to catalyze oxidative modifications to a variety of biological molecules in addition to protein including DNA, RNA and lipid [3, 4]. They target their substrates with similar levels of specificity as other signaling enzymes: Protein hydroxylases generally modify a single residue in a limited number of substrates. Thus far, the types of amino acids targeted for hydroxylation include prolyl, lysyl, asparaginyl, arginyl, aspartyl, and histidyl residues [5–12]. Whilst our understanding of prolyl, lysyl and asparaginyl hydroxylation has been driven by extensive research into their roles in collagen synthesis and hypoxia signaling [7, 10], the biological roles of the other types of protein hydroxylation are less well understood. That being said, it is clear that even poorly characterized hydroxylases are involved in diverse aspects of physiology and in important disease processes [13, 14]. In this article, we focus on summarizing our current understanding of protein histidyl hydroxylation, the main enzymes responsible for catalyzing this novel PTM, and their emerging roles in cell biology, physiology and disease.

Histidyl hydroxylation was first characterized in 2011, with the discovery that factor-inhibiting HIF (FIH), the primary activity of which is asparaginyl hydroxylation, can also catalyze site-specific histidyl hydroxylation in the ankyrin repeat domain of over-expressed Tankyrase-2 [12]. Whether this modification occurs in endogenous Tankyrase-2, and whether it has a physiological role in regulating Tankyrase-2 function, remains unclear. More recently, two closely related 2OG-oxygenases, Myc-induced nuclear antigen (MINA) and nucleolar protein 66 (NO66), were reported to hydroxylate specific histidyl residues in endogenous ribosomal proteins [6]. Therefore, we focus our attention here on MINA and NO66. First, we provide a general background to the wider family of 2OG-oxygenases.

## 2OG-oxygenases

**Fig. 1:**
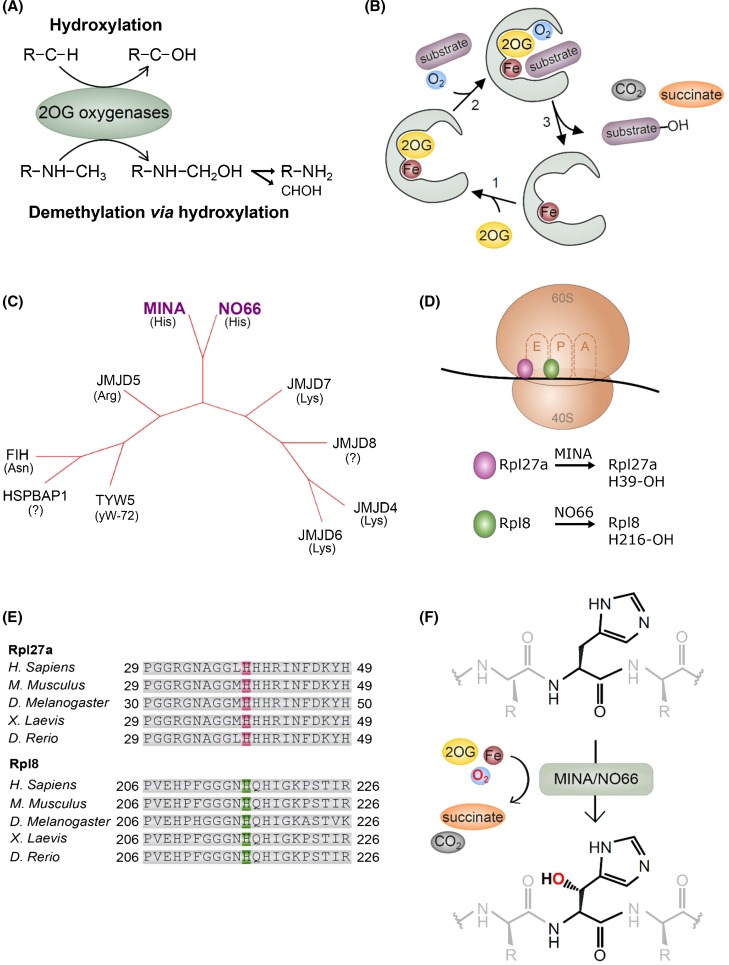
Ribosomal histidyl hydroxylation catalyzed by the 2OG-oxygenases MINA and NO66. **a** 2OG-oxygenases that target protein substrates can catalyze stable hydroxylation (top), or demethylation via a hydroxylation reaction (bottom). Demethylation produces the unmethylated substrates and formaldehyde (CHOH). Note that only demethylation of a mono-methylated amino acid is shown: 2OG-oxygenase-mediated demethylation is also possible at tri- and di-methylated residues. **b** The catalytic cycle of 2OG-oxygenases. For clarity, a graphical representation of only the catalytic pocket, not the whole DSBH domain, is shown. **c** The JmjC-only family of 2OG-oxygenases. Phylogenetic tree constructed using iTOL online software [80] in unrooted tree format using tree data derived from Clustal Omega alignment of the following human protein sequences; MINA (Q8IUF8), NO66 (Q9H6W3), JMJD4 (Q9H9V9), JMJD5 (Q8N371), JMJD6 (Q6NYC1), JMJD7 (POC870), JMJD8 (Q96S16), TYW5 (A2RUC4), FIH (HIF1AN; Q9NWT6) and HSPBAP1 (Q96EW2). Primary biochemical specificities are indicated by His (histidyl hydroxylase), Lys (lysyl hydroxylase), Arg (arginyl hydroxylase), Asn (asparaginyl hydroxylase), yW-72 (hydroxylase of modified Wybutosine nucleoside in tRNAPhe) or ‘?’ (unknown) [6, 9, 11, 81–83]. **d** Ribosomal oxygenases target important functional domains within the ribosome. MINA targets His-39 of Rpl27a within the large (60S) subunit, which is located close to the ‘E’-site, the binding site of the exiting tRNA. NO66 targets His-216 of the 60S subunit protein Rpl8, which is proximal to the peptidyl transferase centre (PTC). The PTC binds to the P- and A-site tRNAs and catalyzes peptide bond formation. **e** The histidyl residues hydroxylated by MINA and NO66 are highly conserved. **f** MINA and NO66 catalyze beta histidyl hydroxylation. NMR studies indicate that hydroxylation occurs on the beta carbon [6]

FIH, MINA and NO66 belong to a gene family of approximately sixty 2OG-oxygenases. These enzymes catalyze various oxidative modifications, including hydroxylation and demethylation (via a hydroxylation reaction) (Fig. 1a), through the action of a common catalytic domain known as a ‘double-stranded beta helix’ (DSBH) [15, 16]. This domain consists of eight anti-parallel beta strands which together form a barrel-like or ‘cupin’ fold. This structure ensures the integration of essential co-factors including Fe-(II), the Krebs cycle intermediate 2OG, molecular oxygen, and the substrate (Fig. 1b) [15, 16]. A conserved ‘2-His/1-Carboxylate’ motif mediates Fe-(II)-binding, whilst the 2OG-binding residues vary across different sub-families.

Catalysis by 2OG-oxygenases initially proceeds via formation of a highly reactive iron (IV)-oxo intermediate (by the oxidative decarboxylation of 2OG), which oxidizes the target substrate (Fig. 1b) [15, 16]. Where a 2OG-oxygenase has a demethylase function, the product formed is an unstable hydroxy-methyl group, which spontaneously fragments producing formaldehyde and the demethylated substrate (Fig. 1a) [13, 17]. The requirement for fundamental nutrients and metabolites means that the activity of these enzymes has the potential to be regulated by changes in the intracellular and extracellular micro-environment. Low tissue oxygen tensions and dysregulation of the Krebs’ cycle have been implicated as important regulators of 2OG-oxygenase function in physiological and disease processes [18, 19]. For example, increased concentrations of the metabolites succinate and fumarate have been shown to competitively inhibit some 2OG-oxygenases [19, 20], and oxygen limitation inhibits hydroxylases that control the proteasomal degradation of the hypoxia-inducible transcription factor HIF [7, 18, 21]. The catalysis of hydroxylation and demethylation by 2OG-oxygenases is described in depth in a number of excellent reviews [2–4, 13, 15–17].

2OG-oxygenases have been implicated in several fundamental cellular processes [4, 14]. Thus, it is perhaps unsurprising that 2OG-oxygenases have been linked with a variety of diseases. These include obesity, cardiovascular and pulmonary disease, neurological abnormalities and cancer [14, 22]. This in turn has stimulated efforts to understand the biochemical activities and cellular functions of 2OG-oxygenases. Indeed, histidyl hydroxylases were initially characterized in disease models, sparking efforts to identify and characterize their substrates and biological functions.

## Initial discovery and characterization of histidyl hydroxylases

MINA and NO66 belong to a subgroup of 2OG-oxygenases whose DSBH has homology to that of ‘Jumonji’, a protein originally named after the cruciform phenotype observed in the corresponding knockout mouse [23]. These DSBH domains, now termed Jumonji-C (JmjC) are present in 2OG-oxygenases that (generally) catalyze protein hydroxylation. The largest phylogenetic group of JmjC hydroxylases catalyze lysyl demethylation of histones and have attracted significant interest for their roles in epigenetics [17]. In addition to the JmjC domain, these proteins also contain functional motifs involved in chromatin biology including ARID, Tudor and zinc finger domains [13].

In contrast, MINA and NO66 belong to a phylogenetically distinct group of JmjC 2OG-oxygenases that do not contain other immediately obvious functional domains, particularly those recognized as having important roles in chromatin biology (see below). As such, this family has been termed the ‘JmjC-only’ 2OG-oxygenase sub-family [17]. There are ten currently identified JmjC-only 2OG-oxygenases in humans (MINA, NO66, JMJD4-8, FIH, TYW5, HSPBAP1 (Fig. 1c), the majority of which are known or predicted protein hydroxylases [4, 13].

MINA was first described in 2002 as a c-myc target gene following gene expression profiling in HL60 cells that conditionally expressed c-myc [24]. Although MINA appears to be ubiquitous [25], its expression does not always correlate with myc levels [26], indicating that other factors also likely contribute. Indeed, SMAD and Sp1 transcription factors have also been identified as regulators of MINA transcription [27]. Consistent with its regulation by growth-responsive transcription factors, MINA levels are induced by mitogens [24], and it is highly expressed in rapidly proliferating tissues such as the testes [28]. Interestingly, MINA levels may also be under the control of stress signaling pathways: Independent gene expression profiling experiments discovered MINA as a gene whose transcription was induced by silica treatment in a Jun kinase-dependent manner (resulting in the label ‘mineral dust-induced gene’, or Mdig) [29, 30].

Proteomic analyses of nucleoli purified from *Xenopus laevis* oocytes also led to the discovery of MINA as a novel nucleolar protein [26]. Eilbracht et al. reported that MINA was localized to the granular component of the nucleoli, a sub-compartment normally associated with late stages of ribosomal biogenesis. Indeed, mass spectrometry analysis of MINA complexes supported its interaction with pre-ribosomal particles [6]. This led to the proposal that MINA may play a role in ribosome biogenesis. Although MINA is enriched in the nucleolus, it is also partially localized to the nucleoplasm [26, 31]. Whether its differential localization reflects multiple cellular functions is unclear, but could be consistent with proposed roles in chromatin biology and transcription (see below).

Interestingly, the biochemical exploration of the nucleolus that discovered MINA also identified a related JmjC-only protein called NO66 [32]. Like MINA, NO66 is also highly conserved through evolution and is ubiquitously expressed [32]. Furthermore, sucrose-gradient experiments demonstrated that NO66 co-fractionates with pre-ribosomal particles, again suggestive of a role in ribosome biogenesis. However, additional localization in nucleoplasmic dots that colocalize with Ki-67, HP1α and PCNA, suggested that NO66 may also have functions in late replicating chromatin [32]. Similar to MINA, these observations may be consistent with independent reports of NO66 controlling chromatin modifications and transcriptional regulation (see below). Indeed, NO66 was independently identified as a member of the myc transcriptional complex and named ‘MAPJD’: Suzuki et al. suggested that transactivation of myc by NO66 could lead to upregulation of genes involved in oncogene signaling and the TGFβ pathway [33].

Overall, the studies outlined above suggested that MINA and NO66 are ubiquitously expressed proteins that have functions in ribosome biogenesis and/or protein translation, and possibly extra-nucleolar functions in genome biology. However, the molecular mechanisms involved in these processes remained unclear, partly because of a lack of understanding regarding the biochemical activities of these enzymes.

## Assigning the biochemical activity of MINA and NO66

Early studies on MINA and NO66 successfully identified the presence of the JmjC domain [24, 26, 32]. Although it was not recognized as catalyzing histone lysine demethylation at that time, the JmjC domain had been proposed to have a role in chromatin remodeling [34, 35]. Subsequently, the assignment of histone lysine demethylase (‘KDM’) activity to some JmjC domains [17] led investigators to test whether MINA and NO66 regulate the methylation status of histone lysine marks. Candidate approaches screened the expression levels of these marks in cells overexpressing MINA or NO66, and led to the assignment of both as KDMs [36, 37], as outlined below.

Lu et al. noted an inverse correlation between MINA and global H3K9me3 levels in lung cancers, and reported that manipulating MINA levels by overexpression or RNA interference caused the anticipated changes in H3K9me3 expression in bronchial epithelial cells [36]. However, a subsequent study by the same group noted marginal effects of MINA on H3K9me3 levels in A549 cells [38]. Interestingly, Chen and colleagues observed that MINA upregulates the expression of KDM4A (a JmjC H3K9me3 demethylase of the KDM family) which they proposed may contribute to the observed regulation of H3K9me3.

A proteomic analysis of Osterix, an osteoblast-specific transcription factor (see below), led to the identification of NO66 as an Osterix repressor [37]. Sinha et al. reported that NO66 regulates Osterix-specific target promoters and proposed that the molecular mechanism involved an unusual H3K4 and H3K36 demethylase activity. Chromatin immunoprecipitation experiments indicated that the chromatin localization of NO66 inversely correlates with H3K4me3 and H3K36me3 levels [39, 40], suggesting that NO66 may regulate the abundance of these epigenetic marks.

Independent, unbiased proteomic screens subsequently identified a distinct biochemical activity for both MINA and NO66. Mass spectrometry analyses of the MINA and NO66 interactomes identified a large number of ribosomal and nucleolar proteins [6], consistent with the work of Eilbracht et al. [26, 32]. Synthetic peptides spanning all these interacting proteins were tested for a +16 Da shift by mass spectrometry, indicative of hydroxylation. These biochemical assays led to the discovery that MINA efficiently targets His-39 of the large ribosomal subunit Rpl27a, and that NO66 is equally effective at modifying His-216 of Rpl8 (Fig. 1d, e) (Rpl27a and Rpl8 are respectively known as uL15 and uL2 under the current naming convention). A subsequent study by an independent group confirmed Rpl8 His-216 hydroxylation by NO66 in vitro [41]. NMR studies confirmed the biochemical assignment and demonstrated that these enzymes hydroxylate at the beta position of the side chain (Fig. 1f) [6]. Endogenous Rpl27a and Rpl8 hydroxylation were dependent on MINA and NO66, respectively, and (importantly), this occurred in a non-redundant fashion. Interestingly, the occupancy of histidyl hydroxylation was extremely high in all healthy cell lines and tissues tested (> 90%), which may suggest a positive role in ribosome biogenesis and/or translation. Structural analyses indicated that Rpl27a His-39 is proximal to the ribosomal E-site tRNA, and His-216 of Rpl8 is close to the peptidyl transferase center (PTC) (Fig. 1d) [42]. Subsequent chemical footprinting analyses of differentially hydroxylated Rpl8 indicated that the NO66 hydroxylation reaction likely serves to properly orientate the 28S rRNA in the vicinity of the PTC, thus ensuring appropriate organization of the PTC in the assembled ribosome [42]. The assignment of ‘ribosomal oxygenase’ activity has recently led to NO66 and MINA being officially named RIOX1 and RIOX2, respectively. Interestingly, the finding that MINA and NO66 are related to YcFD, an *Escherichia coli* JmjC 2OG-oxygenase that catalyzes arginyl hydroxylation of the ribosomal protein Rpl16 [6], suggests that ribosomal oxygenase activity is very highly conserved. The function of MINA, NO66 and YcfD in ribosomal protein hydroxylation is consistent with an emerging role for JmjC-only enzymes, and the wider 2OG-oxygenase family, in protein translation [43].

Are MINA and NO66 ‘bifunctional’ enzymes that possess both ribosomal oxygenase *and* KDM activity? A comprehensive study began to address this question by screening the catalytic domains of 15 JmjC enzymes from across the 2OG-oxygenase family against a panel of histone H3 peptides that spanned each methylation status (me1-3) of four lysyl residues; H3K4, H3K9, H3K27 and H3K36 [44]. These sites included the proposed demethylation targets of both MINA and NO66. Under conditions in which JmjC enzymes of the KDM family catalyzed efficient demethylation of their reported substrates, MINA and NO66 were completely inactive. Importantly, however, under the same conditions, MINA and NO66 catalyzed efficient hydroxylation of Rpl27a and Rpl8 peptides, respectively. Similar findings were independently reported following a focussed study on NO66 [41]. These data would suggest that the primary activity of MINA and NO66 is histidyl hydroxylation.

The specific biochemical activity of an enzyme can also be inferred from detailed structural analysis of its active site. Indeed, structural analyses of JmjC KDMs and hydroxylases have helped to rationalize differences in their biochemical specificities (reviewed in [15]).

## Structural analysis of histidyl hydroxylases

**Fig. 2:**
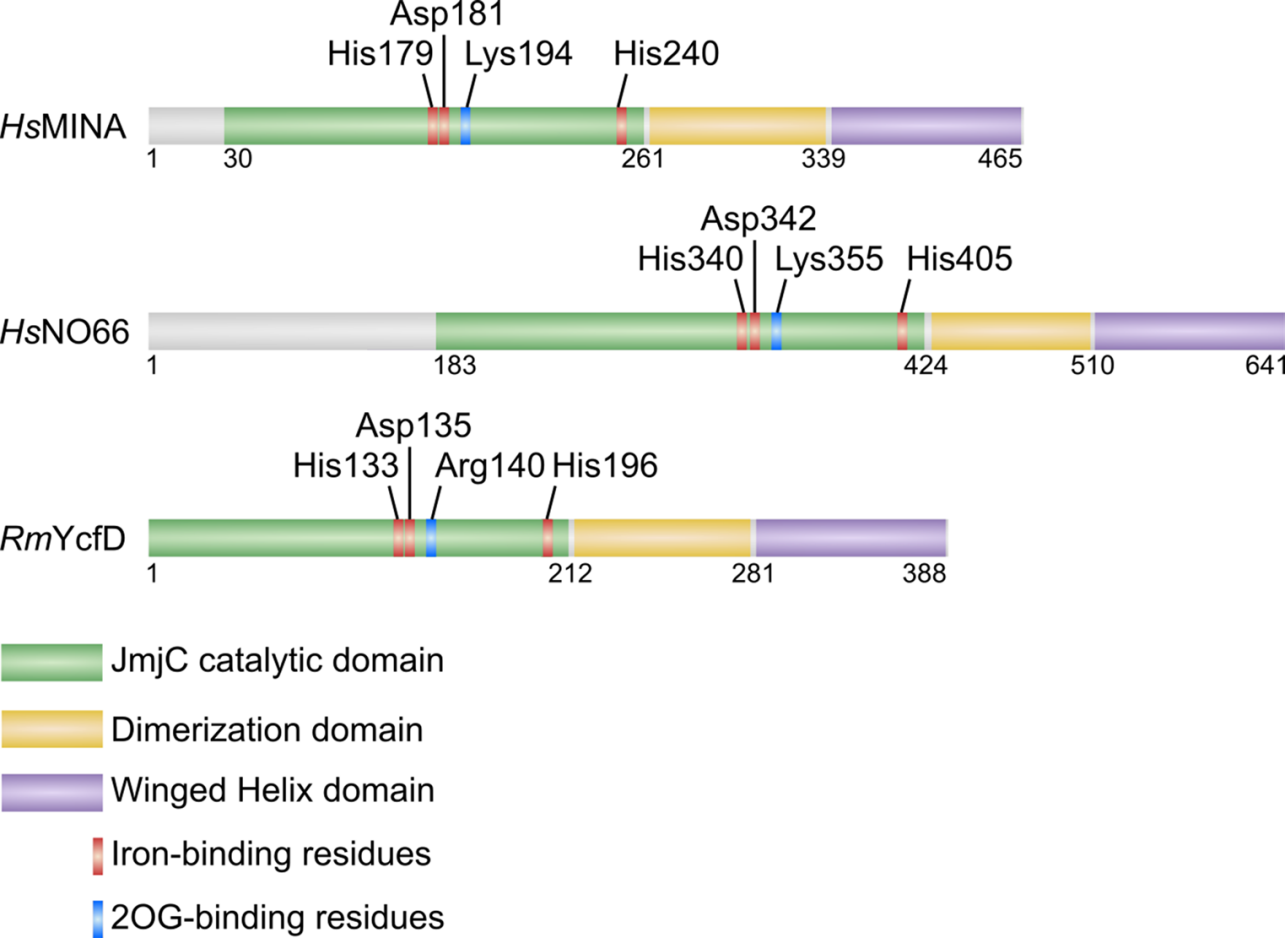
Domain organization of ribosomal oxygenases. Reported crystal structures indicate a unique topology for JmjC ribosomal oxygenases that is conserved from *Homo sapiens* (*Hs*) to prokaryotes (including *R. marinus, Rm*). Note that YcfD catalyzes arginyl hydroxylation of Rpl16 in prokaryotes [6]. Critical catalytic residues are indicated. The dimerization domain is required for oligomerization and activity [45]. The function of the WH domain is unclear but, because of its overall negative charge, is thought unlikely to mediate an interaction with nucleic acid [45]

To investigate the relationship between JmjC ribosomal oxygenases and KDMs, Chowdhury et al. solved the crystal structures of MINA, NO66 and YcfD [45]. Consistent with predictions from primary sequence analyses, these enzymes lacked functional motifs commonly associated with KDMs. Interestingly, however, they all contained a conserved helical motif immediately C-terminal to the JmjC domain that was required for dimerization (Fig. 2). Similar to other JmjC-only hydroxylases [9, 46], oligomerization appears to be required for full RIOX activity [41, 45]. Moreover, MINA, NO66 and YcfD also share another C-terminal fold termed the winged helix (WH) domain (Fig. 2) [41, 45]. Because of the absence of this domain in other 2OG-oxygenases [13] it has been proposed that KDMs likely evolved from RIOXs following the loss of the WH domain [45]. Although its exact function is unclear, the restriction of the WH domain to RIOXs would suggest a highly conserved role in ribosomal protein hydroxylation. WH domains are generally implicated in protein–protein or protein-DNA interactions [47]. However, Chowdhury et al. suggest that the overall negative charge of this domain in MINA, NO66 and YcfD makes direct DNA/RNA-binding unlikely [45]. Therefore, it is possible that the WH domain mediates an interaction with another protein, perhaps a highly conserved factor that targets RIOXs to ribosome biogenesis pathways.

Further evidence supporting that MINA and NO66 are biochemically distinct from KDMs came from detailed structural analyses of their active sites and the binding mode of their substrates [45]. RIOXs bind their substrates in a conserved manner that is similar to related JmjC-only hydroxylases such as FIH, with a specific N- to C- directionality with respect to the catalytic machinery. Importantly, this differs significantly from that of most KDMs, including those considered to be phylogenetically more closely related to MINA and NO66 [45]. Furthermore, there are important differences in how deeply the target side chain binds in the catalytic pockets of RIOXs versus KDMs. Because JmjC-only protein hydroxylases target a carbon in the middle of the side chain, the target residue binds deep within the pocket [45]. In contrast, KDMs target the N^Ɛ^-methyl site at the end of the lysyl residue, so their target residues penetrate less deeply [15]. Finally, the JmjC domains of ribosomal oxygenases also lack two flexible loops within the DSBH that are highly conserved within the KDM subfamily and essential for lysyl demethylase activity. These loops form important interactions with the methylated lysyl side chain to facilitate its accommodation within the active site of the enzyme [15].

Overall, the combined phylogenetic, evolutionary, structural and biochemical data are consistent with the assignment of MINA and NO66 as protein histidyl hydroxylases.

Next, we summarize the current literature describing the emerging roles of these histidyl hydroxylases in diverse aspects of biology and disease.

## NO66 and MINA in physiology and disease

The early studies that led to the discovery and biochemical characterization of MINA and NO66 involved diverse approaches, including enzyme-focussed screens (e.g., MINA/NO66 proteomics), function-specific proteomics (e.g., Osterix), and differential gene expression profiling experiments (e.g. c-myc, silica treatment, and lung cancer). This diversity has, in turn, contributed to an expanding literature on the role of these histidyl hydroxylases in mammalian physiology and disease, as reviewed below.

## NO66

Previous research has identified two main roles for NO66 in physiology and disease, namely skeletal bone formation and tumorigenesis.

### NO66 and skeletal development and ossification

**Fig. 3:**
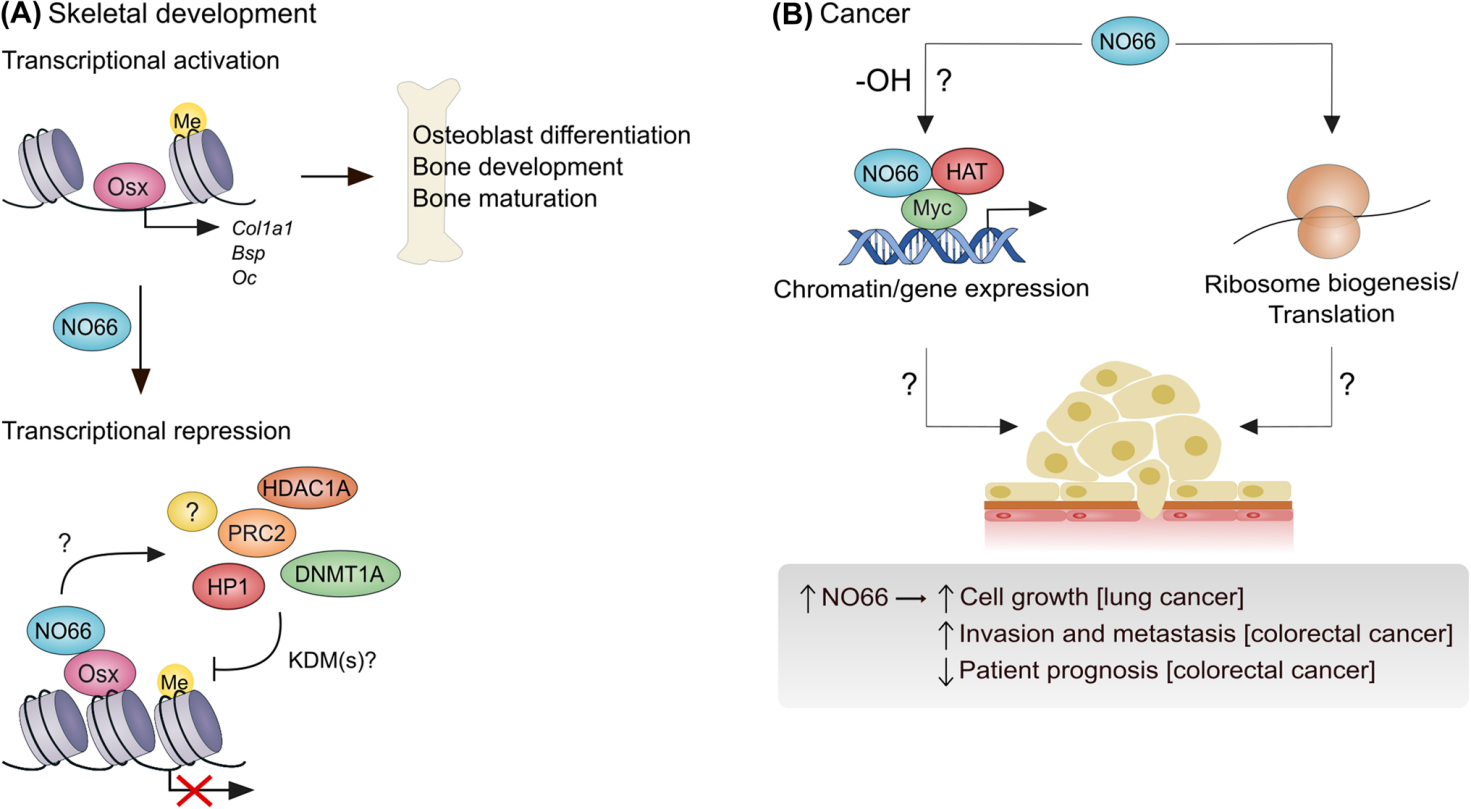
NO66 is implicated in skeletal development and tumorigenesis. **a** NO66 represses the osteoblast-specific transcription factor Osterix (Osx). Osterix drives the transcription of specific target genes (e.g., *Col1a1*) to promote osteoblast differentiation and bone development. The dimerization domain of NO66 interacts with the transactivation domain of Osx, leading to reduced Osx target gene expression. Whether this involves a direct effect of an NO66 histone demethylase activity, or results from the recruitment of multiple epigenetic modifiers (e.g., PRC2, HP1, etc.), is currently unclear (signified by ‘?’). **b** NO66 is over-expressed in lung and colorectal cancers and promotes tumor cell growth and invasion in vitro. Whether the enzymatic activity of NO66 is involved is unclear (‘-OH?’). The molecular mechanisms involved are also unknown, but could be related to roles in myc-driven transcriptional control (left) and/or ribosome biogenesis/translation (right). *HDAC1A* histone deacetylase *1A*; *PRC2* polycomb repressor complex 2; *HP1* heterochromatin protein 1; *DNMT1A* DNA methyltransferase 1A; *Col1a1* collagen type I alpha 1 chain; *Bsp* bone sialoprotein; *Oc* osteocalcin; *HAT* histone acetyltransferase complex (TRRAP and TIP60)

As outlined above, NO66 was detected in a proteomic screen aimed at identifying the interactome of Osterix, an osteoblast-specific transcription factor that is required for osteoblast differentiation and bone formation [37]. Further characterization of this interaction identified that NO66 inhibits the ability of Osterix to drive osteoblast-specific gene transcription (Fig. 3a), through an interaction between the dimerization domain of NO66 and a defined 16 amino acid sequence within the transactivation domain of Osterix [37, 48]. Overexpression of NO66 is sufficient to suppress the transactivation domain of Osterix using in vitro cell models [37], and mesenchymal-specific overexpression inhibits osteoblast proliferation and differentiation, skeletal growth and bone formation in mice [49]. Conversely, mesenchymal-specific ablation of NO66 increases the expression of genes that promote bone growth and development in mice, with a consequent increase in bone mass and density [50].

Importantly, overexpression experiments in HEK293T cells suggested that the ability of NO66 to repress the transactivation domain of Osterix is dependent on its enzymatic activity [37]. Although de Crombrugghe and colleagues suggest that NO66 regulates Osterix target genes via an intrinsic histone demethylase activity [37, 40], this may require further consideration, for reasons outlined above [15, 41, 44, 45]. Interestingly, other work suggests that NO66 exists in complexes containing important epigenetic modifiers including DNA methyltransferase 1A (DNMT1A), histone deacetylase 1A (HDAC1A), HP1 and PRC2 (Fig. 3a) [39, 40], raising the possibility that NO66 might regulate histone methylation status and target gene expression via an intermediary.

Whilst the molecular mechanism by which NO66 influences bone formation in vivo and osteoblast differentiation in vitro remains unclear (Fig. 3a), it is evident that NO66 is an important regulator of bone growth and mineralization (a mechanistic discussion of the roles of NO66 (and MINA) is presented at the end of this review). Considering the potential implications for diseases such as osteoporosis and osteopenia [51], further research in this area is warranted.

### NO66 and cancer

2OG-oxygenases are attracting significant academic and pharmaceutical interest for their roles in tumor development and metastasis [14]. These roles are diverse and include, but are not limited to, cellular dedifferentiation via histone demethylation, hypoxia sensing in tumors, and regulation of inflammation and resistance to apoptosis [13, 14, 18]. In addition to aberrant expression and genetic alteration, 2OG-oxygenases may be deregulated by additional mechanisms in cancer, including competitive inhibition by ‘onco-metabolites’ (e.g., fumarate, see Introduction), and by low oxygen tensions in poorly vascularized areas of the tumor [13, 18–20]. Although it is currently unclear whether such mechanisms regulate MINA and NO66 activity in tumors, there is some evidence suggesting that ribosomal hydroxylation may be reduced in severe hypoxia. Ribosomal oxygenases are some of the only 2OG-oxygenases downregulated at the transcriptional level during hypoxia [52]. Indeed, reduced NO66 protein expression under severe hypoxia correlated with impaired Rpl8 hydroxylation [6]. Whether this effect was primarily due to reduced enzyme abundance, and/or a direct effect on enzyme activity remains unclear. Furthermore, it is not yet known whether MINA and NO66 ‘sense’ oxygen in the physiological range in a manner akin to the HIF hydroxylases, and whether such effects are relevant to tumor biology. That being said, there is mounting evidence supporting the role of both enzymes in cellular processes implicated in cancer.

The first indication that NO66 may have a role in cancer followed comparative gene expression profiling in normal versus non-small cell lung cancer (NSCLC) [33]. Suzuki et al. identified NO66 mRNA as frequently over-expressed in the majority of NSCLC samples and lung cancer cell lines tested. Overexpression of NO66 conferred increased growth potential upon NIH3T3 fibroblasts, whilst RNA interference in LC319 and A549 lung cancer cells reduced growth. Of interest with respect to the function of NO66 in transcriptional regulation, and the potential role of MINA downstream of Myc, Suzuki et al. also characterized transcriptional activation mediated by a complex containing NO66, Myc and histone acetyltransferases (Fig. 3b) [33].

In addition to the cell biology approaches outlined above, pathology analyses have investigated the expression of NO66 in human tumor samples. High levels of NO66 in colorectal cancer samples were associated with metastatic potential, venous invasion and lymph node metastasis: Patients whose tumors stained positively for NO66 had significantly shorter survival and disease-free survival [53]. Similar to the work in lung cancer cell lines described above, overexpression of NO66 in colorectal cells was also sufficient to promote their growth, and their invasion (Fig. 3b).

The mechanism(s) by which NO66 promotes tumor cell growth and invasion remains unclear. Further work is required to determine the relative contribution of Rpl8 hydroxylation versus other potential functions in gene expression control and epigenetics (discussed in more detail below).

## MINA

MINA has generally been studied in more detail than NO66 and is implicated in a wider range of physiological and pathological processes, particularly those related to the immune system and cancer.

### MINA and cancer

**Fig. 4:**
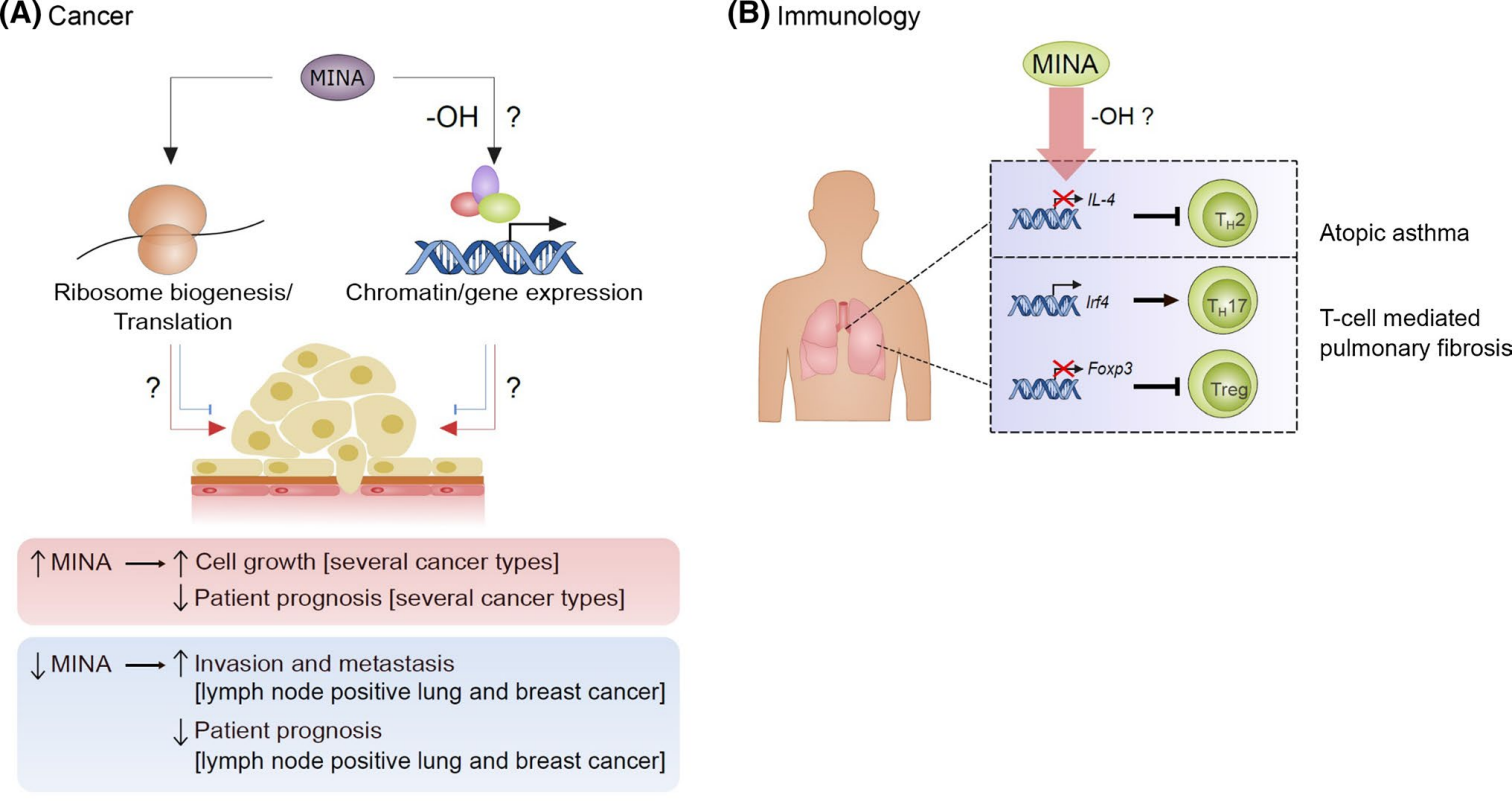
MINA is implicated in tumorigenesis and T-cell differentiation. **a** MINA is over-expressed in multiple tumor types and is required for cancer cell proliferation in vitro. Whether the enzymatic activity of MINA is required is unclear, particularly with respect to potential roles in gene expression control (‘-OH’?). ‘Driver’ roles for MINA in cancer are denoted by red arrows. MINA may also have tumor suppressor activity in some contexts, possibly via regulation of invasion/metastasis (denoted by blue ‘flat head’ arrows). These paradoxical effects were first uncovered by pathology analyses in lung and breast cancer (summarized at the bottom). **b** Immunology: MINA regulates the differentiation of specific T-cell subsets in asthma and pulmonary fibrosis. MINA represses Interleukin-4 (IL-4) transcription to regulate T-helper 2 (T_H_2) cell bias (top). MINA upregulates a T-helper 17 (T_H_17) differentiation transcriptional program and suppresses the expression of the master regulator of regulatory T-cell (Treg) differentiation (Foxp3)

The identification of MINA as target of the myc proto-oncogene (see above) led to pathology studies aimed at detecting its protein expression in a range of tumor types by immunohistochemistry. There are now several reports describing MINA overexpression in multiple cancer types including colorectal [54], lung [30, 55], esophageal [56], lymphoma [57], cholangiocarcinoma [58], gingival squamous cell carcinoma [59], neuroblastoma [60], liver [61, 62], gastric [63], pancreatic [64], multiple myeloma [65], and breast cancers [66]. Importantly, MINA expression was associated with poor prognosis in some, but not all, of these studies [56, 60, 63, 65–67]. Interestingly, MINA overexpression positively correlated with immunohistochemical markers of proliferation in several studies [58–60, 62, 67]. These observations, in combination with MINA being a growth-responsive gene (see above) [24], led to the proposal that MINA may support tumorigenesis by promoting cancer cell proliferation. Indeed, cell biology studies have confirmed that blocking MINA expression (using RNA interference) limits the proliferative capacity of several tumor cell lines in vitro [24, 54, 56, 61, 64, 68]. Together, these data would indicate that MINA expression supports the proliferative drive of tumor cells (Fig. 4a), at least in some contexts.

Recent work suggests that the role of MINA in cancer may be more complex than first thought, however. Specifically, MINA overexpression may not always correlate with *poor* patient prognosis. Although MINA overexpression was associated with worse survival in lung cancer patients who were staged ‘lymph node negative’ (N0) or ‘possible proximal lymph node metastasis’ (N1) (consistent with the cancer ‘driver’ work reviewed above), MINA expression was associated with *improved* prognosis in patients with distant lymph node invasion (N2) or ‘distant metastasis likely’ (M1) [31]. This observation might suggest that MINA has opposing roles at different stages of tumorigenesis, with a growth-promoting role in early cancer, and a tumor suppressor role in later stage disease, possibly via modulating invasion and/or metastasis (Fig. 4a). Perhaps consistent with the latter, overexpression of MINA suppressed the migration and invasion of A549 and H226B lung cancer cells in vitro [31, 69]. Conversely, MINA knockdown enhanced their invasion and migration [31, 69], and in a manner that was associated with changes in markers of epithelial–mesenchymal transition [70].

Importantly, the complex association of MINA expression with prognosis may not be restricted to lung cancer. Breast cancer patients without lymph node involvement are reported to have worse survival if their tumors express higher levels of MINA, whereas MINA expression is associated with improved survival of patients that are lymph node positive [66]. This, together with the studies outlined above, might suggest that MINA can have a tumor suppressor function in some contexts, possibly via the control of invasion and migration. However, it remains unclear whether MINA is a *bona fide* tumor suppressor gene, and if so, what the molecular mechanisms involved might be (Fig. 4a) (further discussed below).

### MINA and immunology

The first evidence implicating MINA as an immuno-modulator came from mouse studies in which it was identified as a genetic determinant of ‘T-helper type 2 (T_H_2) bias’ [71], the tendency of naive CD4^+^ T cells to differentiate into IL4-producing T_H_2 cells. T_H_2 bias is an important immunological process that regulates susceptibility to autoimmune, allergic, and infectious disease [72]. Okamoto et al. reported that MINA modulates T_H_2 bias by acting as a direct repressor of IL-4 transcription (Fig. 4b) [71].

An independent study subsequently implicated MINA in controlling the balance between additional T cell sub-types, specifically Th17 and Treg cells [73]. Th17 cells are pro-inflammatory IL17-producing T-cells that have important roles in protecting against extracellular pathogens, and in the onset of autoimmune diseases [74]. Yosef et al. undertook a temporal analysis to identify the dynamic regulatory gene expression network involved in Th17 differentiation, and studied the role of novel targets in these networks using nanowire-based siRNA delivery [73]. This approach identified that MINA mRNA is strongly induced during Th17 differentiation, and that MINA promotes the expression of Th17 factors such as ROR-γt, Batf, and Irf4, whilst suppressing the expression of the Foxp3 transcription factor, which plays a pivotal role Treg in biology (Fig. 4b).

The two studies outlined above suggested, for the first time, that MINA has potentially important roles in regulating T-cell differentiation, and that it might, therefore, be involved in the etiology of some autoimmune, allergic, and infectious diseases. Indeed, recent evidence implicates MINA in asthma and pulmonary fibrosis. A case–control study in a Han-Chinese population identified a single-nucleotide polymorphism (SNP) of the MINA gene that was associated with a significant increase in the risk of atopic asthma, and that patients heterozygous for this allele had elevated serum levels of IgE and IL-4 (the effect on MINA expression was not reported) [75]. Interestingly, a recent study using a mouse model of asthma induced by house dust mite aeroallergens found that MINA knockout significantly ameliorated airway hyper-responsiveness and decreased inflammation [76]. Counter to the role of MINA in repressing IL-4 in naive CD4^+^ T cells (as above), this MINA knockout phenotype was associated with *decreased* levels of IL4. The authors speculated that this may be mediated via an independent role for MINA in promoting T_H_2 cytokine expression in antigen presenting cells. Others have proposed that the reduced airway disease in this model may be associated with a defective Th17 response [77]. If correct, this would suggest that the known role of MINA in Th17 differentiation (outlined above) may be dominant with respect to its role in T_H_2 bias, at least in this particular disease. This hypothesis may be supported by a recent study in which heterozygous knockout of the MINA gene in mice was associated with reduced silica-induced pulmonary fibrosis, possibly due to an observed decrease in the Th17 response (and corresponding increase in the Treg response) [78].

In summary, the finding that MINA influences the balance between multiple T-cell subsets may be consistent with important roles in several T-cell-mediated responses and diseases, including perhaps other pathological contexts such as transplant rejection, autoimmunity and infection. The key now is to understand the determinants that influence the role of MINA in T-cell biases and specific immunological disease states. In addition, further studies are needed to shed light on the molecular mechanisms by which MINA exerts its complex effects on the immune system.

## What are the molecular mechanisms under-pinning the role of MINA and NO66 in physiology and disease?

In the preceding sections of this review, we have summarized the current literature on the protein histidyl hydroxylases MINA and NO66, including reports of diverse roles in fundamental cellular pathways and important physiological and pathological processes. Although the biomedical importance of these enzymes is becoming increasingly clear, the molecular mechanisms involved, and how these might be deregulated in disease, have not yet been established. In the future it will be important to determine whether the enzymatic *activity* of these 2OG-oxygenases is required for any of the functions described. It is possible, for example, that non-enzymatic functions may be involved, perhaps mediated via the WH domain. If enzyme activity is required, however, it will be of interest to determine how hydroxylation regulates substrate function to elicit the corresponding biological outcome. It will be worthwhile considering whether Rpl27a and Rpl8 modification are likely to solely account for the diversity in biological functions reported. If histidyl hydroxylation functions in ribosome biogenesis to promote increased rates of bulk protein synthesis, it could perhaps explain the role of these enzymes in supporting cell growth, and the association of their expression with poor cancer prognosis (Figs. 3b, 4a). However, it is perhaps more difficult to conceptualize how increased ribosome biogenesis could elicit biological specificity in terms of transcriptional control (e.g., MINA and IL17/Foxp3, Fig. 4b) or physiology (e.g., NO66 and bone development, Fig. 3a). Such specificity might be more easily rationalized if the function of ribosomal protein hydroxylation was to control the translation of specific mRNAs, as opposed to bulk ribosome biogenesis. The former could, perhaps, be consistent with the localization of MINA- and NO66-catalyzed histidyl hydroxylations to functionally important sites of the ribosome (Fig. 1d). Of course, other explanations are also possible, and these may not necessarily be mutually exclusive. An intriguing possibility is that other, as yet unidentified substrates exist for these protein hydroxylases. Considering the biology reviewed above, one might predict that such novel targets could have functions in transcription and/or chromatin biology/epigenetics (Figs. 3, 4). Thus, further study of the role of MINA and NO66 histidyl hydroxylase activity is warranted. Future findings will be critical for not only providing mechanistic insight into how these enzymes regulate their respective physiological processes and disease, but also for determining whether the development of small molecule inhibitors is merited. Importantly, competitive inhibitors of 2OG-oxygenases are in clinical trials for other indications [79], suggesting that targeting MINA and/or NO66 is theoretically possible. Such inhibitors would likely benefit from stringent enzyme specificity considering the role of other 2OG-oxygenases in fundamental cellular processes [4, 14, 22]. Bearing in mind the paradoxical roles of MINA in cancer (Fig. 4b) [31, 69], the contexts of their potential medical applications should also be very carefully considered.

## Concluding remarks

Here, we have reviewed our current understanding of the role of the histidyl hydroxylases MINA and NO66 in cell biology, homeostasis and disease. We hope to have highlighted that these enigmatic enzymes have intriguing roles in fundamental cellular processes that warrant further investigation. In particular, this review emphasizes the need for future mechanistic studies that clarify the role of hydroxylase activity, and the identity of critical targets, in the cellular pathways and diseases discussed. Such work will be necessary to build a solid foundation from which to efficiently and successfully translate these 2OG-oxygenases into the clinic. Finally, it remains to be seen whether the proteome contains hydroxy-histidyl residues deposited by other protein hydroxylases. These could include enzymes from novel classes, or perhaps the biochemically unassigned members of the JmjC-only family (Fig. 1c). It is intriguing to consider the possibility that MINA and NO66 might represent the ‘tip of the iceberg’ with respect to the biology of histidyl hydroxylation.

## Abbreviations

2OG: 2-Oxoglutarate
MINA: Myc-induced nuclear antigen
NO66: Nucleolar protein 66 kilodalton
HIF: Hypoxia-inducible factor
FIH: Factor-inhibiting HIF
DSBH: Double-stranded beta helix
JmjC: Jumonji-C
RIOX: Ribosomal oxygenase
KDM: Histone lysine demethylase
